# Whole genome analyses based on single, field collected spores of the arbuscular mycorrhizal fungus *Funneliformis geosporum*

**DOI:** 10.1007/s00572-022-01091-4

**Published:** 2022-09-26

**Authors:** Shadi Eshghi Sahraei, Marisol Sánchez-García, Merce Montoliu-Nerin, David Manyara, Claudia Bergin, Søren Rosendahl, Anna Rosling

**Affiliations:** 1 Department of Ecology and Genetics, Uppsala University, Uppsala, Sweden; 2 Department of Forest Mycology and Plant Pathology, Uppsala Biocentre, Swedish University of Agricultural Sciences, Uppsala, Sweden; 3 Department of Cell and Molecular Biology, Uppsala University, Uppsala, Sweden; 4 Microbial Single Cell Genomics Facility, Department of Cell and Molecular Biology, Science for Life Laboratory, Uppsala University, Uppsala, Sweden; 5 Department of Biology, University of Copenhagen, Copenhagen, Denmark

**Keywords:** AM fungi, Single spore, Morphology, Phylogeny, rDNA, Single nucleus sequencing

## Abstract

Arbuscular mycorrhizal (AM) fungi are ubiquitous mutualistic symbionts of most terrestrial plants and many complete their lifecycles underground. Whole genome analysis of AM fungi has long been restricted to species and strains that can be maintained under controlled conditions that facilitate collection of biological samples. There is some evidence suggesting that AM fungi can adapt to culture resulting in phenotypic and possibly also genotypic changes in the fungi. In this study, we used field isolated spores of AM fungi and identified them as *Funneliformis geosporum* based on morphology and phylogenetic analyses. We separately assembled the genomes of two representative spores using DNA sequences of 19 and 22 individually amplified nuclei. The genomes were compared with previously published data from other members of Glomeraceae including two strains of *F. mosseae*. No significant differences were observed among the species in terms of gene content, while the single nucleotide polymorphism density was higher in the strains of *F. geosporum* than in the strains of *F. mosseae*. In this study, we demonstrate that it is possible to sequence and assemble genomes from AM fungal spores sampled in the field, which opens up the possibility to include uncultured AM fungi in phylogenomic and comparative genomic analysis and to study genomic variation in natural populations of these important plant symbionts.

**Supplementary Information:**

The online version contains supplementary material available at 10.1007/s00572-022-01091-4.

## Introduction

Arbuscular mycorrhizal (AM) fungi form symbiotic interactions with the vast majority of plant species thus forming integral parts of terrestrial ecosystems (Parniske 2008; Smith and Read 2010). Plants benefit from the interaction with AM fungi through improved mineral nutrient and water uptake as well as increased stress tolerance. On the other hand, the AM fungi are obligate symbionts and obtain all their carbon and energy from their plant partners (Smith and Read 2010). Despite generally broad host ranges with little to no host preference (Bonfante and Genre 2010), we know that the species composition of AM fungal communities has functional consequences for terrestrial ecosystems (Hoeksema et al. 2018; Koziol et al. 2018). However, it is important to remember that most experimental studies exploring the biology of symbiosis are limited to AM fungi that can be maintained in culture, either in pots with their host plants or in vitro with root-organ cultures. Most AM fungi complete their life cycles underground, and their multinucleate asexual spores often are the largest biological unit that can be collected for morphological characterization and sequence identification (Bonfante and Genre 2010). Their biology has constrained whole genome analysis of AM fungi, and many studies have focused only on those species and strains from which ample biological material can be obtained (Fortin et al. 2002; Abdellatif et al. 2019).

As a consequence of cultivability, species and strains in the genus *Rhizophagus*, which can be grown in vitro in root-organ cultures, are among the genomically most well-studied AM fungi (Kobayashi et al. 2018; Lin et al. 2014; Morin et al. 2019; Tisserant et al. 2013; Yildirir et al. 2022). Genomes have also been successfully sequenced and assembled from representatives of the order Diversisporales, such as *Diversispora epigaea* which is an AM fungus that forms above-ground sporocarps from which genome assemblies can be generated using metagenomic approaches (Sun et al. 2019) as well as two species in Gigasporales (Morin et al. 2019; Venice et al. 2020). Data from these important contributions have together demonstrated systematic features that distinguish the AM fungal genomes from their closest sister lineages. Together with the cyanobacteria symbiotic fungus *Geosiphon pyriformis*, AM fungi appear to have lost much of the ability to break down plant cell walls, in addition to genes involved in the synthesis of fatty acids and thiamine metabolism (Kobayashi et al. 2018; Malar et al. 2021). Future studies may shed light on gains and losses of specific genes in AM fungal lineages as taxon sampling has dramatically increased with recent development of genome assembly workflows from single nucleus sequencing (Montoliu-Nerin et al. 2020) making genome assemblies from species across seven families of AM fungi available (Montoliu-Nerin et al. 2021).

Interestingly, meta-analyses of soil community composition suggest that the AM fungal taxa that can be maintained in cultures are overrepresented in disturbed ecosystems and that wild plants host fewer cultured taxa than domesticated plants (Ohsowski et al. 2014). These observations indicate that ruderal life history strategies likely are overrepresented among AM fungal taxa used in controlled experimental studies and whole genome analyses (Ohsowski et al. 2014). Some AM fungi can be grown in pot culture for extended periods of time. For example, the most well-sequenced strain of *R. irregularis* (Tisserant et al. 2013; Yildirir et al. 2022) has been maintained in laboratory conditions since the 80 s (Stockinger et al. 2009). Emerging evidence suggests that growth under controlled conditions influences AM strains by selecting phenotypes that perform well under those conditions (Kokkoris and Hart 2019; Kokkoris et al. 2019). This highlights the importance of expanding whole genome analyses of AM fungi to ecologically relevant species and strains.

High levels of intra-specific genomic and phenotypic variation have been reported for AM fungi, including examples from the genus *Funneliformis* (Munkvold et al. 2004). Based on genetic markers, variation also has been observed within morpho-species assigned to *Funneliformis*, collected from fallow and cultivated soils (Rosendahl and Matzen 2008). The genomic structure of genetic variation in AM fungi remains well studied; however, only in *R. irregularis* for which population sampling (Koch et al. 2004) has allowed the discovery of mating type linked heterokaryosis (Ropars et al. 2016). Furthermore, chromosome level assemblies has revealed genome structures with compartments of different variability (Yildirir et al. 2022). The aim of this study was to test if whole genome assemblies suitable for phylogenomic analysis could be generated from individually amplified and sequenced nuclei isolated from AM fungal spores collected from the field. With these assemblies in hand, we also explored within-genus differences regarding gene content and density of single nucleotide polymorphisms (SNPs).

## Methods and materials

A single spore genome sequencing workflow developed by Montoliu-Nerin et al. (2020) was applied to spores from a field sampled morphotype of an AM fungus. Two spores were selected for nuclei isolation, sorting, amplification, and sequencing to assemble reference genomes as described below. Assemblies were used for species identification confirmation with established genetic markers. We then performed phylogenomic and comparative genomic analyses, estimates of SNPs, and characterization of the mating type (MAT) locus.

### Field sampling and morphotyping of spores

A *Potentilla* sp. specimen with attached soil was collected using a hand shovel, at the Kungsängen Nature Reserve, N59 50′, E17 40′ (Uppsala, Sweden), on September 17th, 2019. This protected grassland is managed by grazing and the site is most famous as home to the largest population of *Fritillaria meleagris* in Sweden. The sample was stored at + 4 °C until the next day when soil (almost 30 g) was blended with 0.5L of tap water. After three pulses of blending, the suspension was separated through a set of sieves with sequentially different pore sizes (1 mm, 500 µm, 200 µm, and 38 µm) and rinsed with tap water. Particles from the 200-µm and 38-µm sieves were collected and transferred to Falcon tubes containing 20 mL of 60% sucrose solution. The tubes were centrifuged for 1 min at 3000 rpm and the supernatant was passed through a 5-cm diameter sieve with 38-µm pore size and rinsed thoroughly with tap water. The contents of the sieve were transferred to a petri dish for morphological examination under a Model SMZ800 stereomicroscope (Nikon, Japan). A number of spores identified as belonging to the genus *Funneliformis* (morpho-species *F. geosporum*) were separated on a moist filter paper (Fig. S1). Some of the spores were examined under an Olympus BX41 microscope (Olympus corporation, Tokyo, Japan), to confirm morphotype identification. Remaining spores were transferred to an Eppendorf tube and washed with distilled water.

### Single nuclei sorting, amplification, and sequencing

Two spores of the *F. geosporum* were placed in separate 1.5-mL tubes with 150 μL of 1 × PBS (hereafter referred to as *F. geosporum* (A) for field collected spore A and *F. geosporum* (B) for field collected spore B). Then, spores were crushed with a sterile pestle and stained with 1 μL of 200 × SYBR Green I Nucleic Acid stain (Invitrogen™, Thermo Fisher Scientific, MA, USA). Nuclei sorting was performed in accordance with a previously established protocol (Montoliu-Nerin et al. 2020) in which the solution was immediately transferred to a 0.5-mL tube to allow the debris to settle during the 30–60 min of staining. The nuclear sorting was performed at the SciLifeLab Microbial Single Cell Genomics Facility with a MoFlo™ Astrios EQ sorter (Beckman Coulter, USA) as in Montoliu-Nerin et al. (2020, 2021). From each spore, individual nuclei were isolated into 48 wells of one 384-well plate. Plates were stored at − 80 °C until whole genome amplification with the enzyme Phi29 via multiple displacement amplification (MDA) was done, under clean conditions, using the Repli-g Single Cell kit (Qiagen) in a 10-μL reaction volume. The nucleic acid stain SYTO 13 was added to the reaction in order to monitor the DNA amplification over time. We performed a PCR amplification of rDNA markers on a 1:10 dilution of all amplified nucleus samples, using fungal and bacterial specific primers and following the protocol in Montoliu-Nerin et al. (2020). For each of the two isolates, a total of 22–24 amplified nuclei samples that tested positive for fungi and negative for bacteria were selected for sequencing (Supplementary datafile 1). Library preparation and sequencing was done at the SNP and SEQ Technology Platform in Uppsala at the National Genomics Infrastructure (NGI) Sweden and Science for Life Laboratory (SciLifeLab), using the TruSeq PCR free DNA library preparation kit (Illumina Inc.), followed by 150 cycles of paired-end sequencing using the NovaSeq6000 system and v1 sequencing chemistry (Illumina Inc.).

### Genome assembly

A total of 22 and 19 amplified nuclei were successfully sequenced from field collected spores A and B, respectively (Table S1). Reads obtained from the amplified nuclei were combined and then normalized using bbnorm of BBMap v.38.08 (Bushnell 2014, 2015) with a set average depth of 100 ×. Thereafter, SPAdes v.3.12.0 (Bankevich et al. 2012) was used to generate one genome assembly per spore. Assembly statistics were obtained with QUAST v.5.0.2 (Gurevich et al. 2013), and genome completeness was estimated using BUSCO v.3.0.2b with the fungi_odb9 as lineage setting, and *rhizopus_oryzae* as species set (Simão et al. 2015). To confirm spore identification to genus, the extracted SSU region was combined with the taxon rich SSU alignment from Krüger et al. (2012) and SSU regions extracted from previous AM genomes sequenced by our team (Montoliu-Nerin et al. 2021). To further confirm identity of the spores, phylogenetic analyses were performed on informative genomic regions with available reference data, including the D2 region of rDNA large subunit (LSU), intron 1 from FOX2, and intron 4 from TOR2. These regions were extracted from the assembly using BLASTn v.2.11.0 (Camacho et al. 2009). These markers have been characterized and used in previous studies of genetic variation among AM fungi (Krüger et al. 2012) and within the genus *Funneliformis* (Stukenbrock and Rosendahl 2005). FOX2 encodes a protein involved in peroxidal β-oxidation (Requena et al. 1999), and TOR2 encodes a protein involved in cell cycle processes (Requena et al. 1999). To resolve species identity of the spores, the extracted sequences were aligned with known *Funneliformis* sequences from GenBank and from local sequence resources. The alignment was visually inspected in AliView (Larsson 2014) before reconstructing a maximum likelihood (ML) phylogeny with 1000 bootstrap replicates using IQ-TREE v.2.0 (Minh et al. 2020).

### Searching for a putative MAT-locus

To investigate the presence of the putative MAT-locus in the *Funneliformis* reference genome assemblies, we used the MAT-locus nucleotide sequence of *R. irregularis* (GenBank KT946687) as query in a BLASTn search in all *Funneliformis* assemblies. Requiring an e-value of zero identified two contigs in each of the four *Funneliformis* genome assemblies. The hit contigs were aligned with the query sequence and visually inspected in Geneious 11.0.5 (Biomatters Ltd). Only one contig from each assembly contained a possible homologue for one of the flanking regions (choline transporter-like protein) of the MAT-locus as annotated in KT946687. These four contigs were trimmed to cover the putative choline transporter-like homologue and regions that aligned within species. With the aim to determine if the analyzed *Funneliformis* genome assemblies represented strains that were homokaryotic or heterokaryotic for the MAT-locus (Ropars et al. 2016), we generated single nucleus genome assemblies and queried these with their trimmed contig including the putative choline transporter-like homologue. All single nucleus samples were individually assembled following the workflow for single nuclei assembly as described in Montoliu-Nerin et al. (2020). From the single nuclei assemblies, all contigs with a BLASTn hit score of zero were then aligned to the query and visually inspected using Geneious.

### Taxon sampling

Based on the phylogenetic placement of *F. geosporum* (field collected spores A and B) using different gene regions (SSU, D1 of LSU, TOR2, and FOX2) extracted from the assemblies (Figs. S2–S5), we performed phylogenomic analyses of Glomeraceae. For this, we included six previously published genome assemblies of taxa within the family. Two published *F. mosseae* were included, of which the strain UK2014 was initially listed as *F. caledonius* but has been reidentified as *F. mosseae* (W. Wheeler personal communication) in line with phylogenomic analyses (Montoliu-Nerin et al. 2021). The genus *Rhizophagus* was represented by two published assemblies of the *R. irregularis* strain DAOM197198 (Chen et al. 2018; Montoliu-Nerin et al. 2021) and one assembly of the *R. irregularis* strain DAOM229456 (Morin et al. 2019). The latter was previously published under the name *R. diaphanus* (Morin et al. 2019), but phylogenetic analyses suggest that this strain actually belongs to *R. irregularis* (Błaszkowski et al. 2018). The genus *Oehlia* is represented by *O. diaphana* strain DAOM227022. A genome assembly of this strain was previously included in phylogenomic analyses under the name *R. cerebriforme* (Malar et al. 2021; Montoliu-Nerin et al. 2020; Morin et al. 2019), but based on molecular and morphological data, this strain was shown to represent *O. diaphana* (Błaszkowski et al. 2018). In the current study, names of all included taxa were corrected and updated in accordance with the latest taxonomy for these lineages (Błaszkowski et al. 2018) (Table S2). Based on a previous phylogenomic analysis of AM fungi (Montoliu-Nerin et al. 2021), we used published genome assemblies representing three taxa from the Diversisporales, and *Claroideoglomus candidum* as outgroup (Table S2).

### Gene prediction

Gene prediction was performed on all genome assemblies generated from single nucleus sequencing with an in-house fungal annotation pipeline v.4.0, available at https://bitbucket.org/scilifelab-lts/genemark_fungal_annotation/src/master/. The pipeline first uses RepeatModeler v.1.0.8 (Smit and Hubley 2015) to de novo predict repeat elements from each assembly before masking repeats from the genome assembly with RepeatMasker v.4.0.7 (Smit et al. 2015). The pipeline then uses MAKER v.3.01.1-beta (Cantarel et al. 2008) to align the protein sequences in UniProt/Swiss-Prot (Bateman et al. 2021; UniProt Consortium 2018) to the masked genome assembly. Then, it uses GeneMark-ES v.4.33-es (Ter-Hovhannisyan et al. 2008) to de novo predict protein coding genes from the masked genome assemblies, guided by the locations of the aligned proteins from UniProt/Swiss-Prot. A minimum contig size of 10 Mb was calculated for each assembly to be included in the self-training process of the GeneMark algorithm. For published genome assemblies, the gene predictions were included as published (Chen et al. 2018; Morin et al. 2019; Montoliu-Nerin et al. 2021).

### Functional annotation

Predicted genes from all included genomes were functionally annotated using FunAnnotate v.1.8.9 (Palmer and Stajich 2020). Four databases were used to annotate the gene content, including InterProScan (Jones et al. 2014) for functional domain annotation, MEROPS v.12 (Rawlings et al. 2014) for peptidases, SignalP v.4.1f (Nielsen 2017) for secreted proteins, and dbCAN v.9 (Yin et al. 2012) to identify carbohydrate activated enzymes also known as CAZymes. For each genome assembly, the Pfam domains were normalized by the total number of identified domains in each taxon using the “decostand” function in the vegan package v2.5–7 (Oksanen et al. 2020). Domain composition among included genomes was then visualized using non-metric multi-dimensional scaling (nMDS) based on Bray–Curtis dissimilarity. For the other datasets, predicted genes in each gene family were normalized to the total number of predicted genes in each assembly (Table S3).

### Phylogenomic and comparative analyses

Based on the amino acid sequences of predicted genes, we identified single copy orthologs (SCOs) present in all taxa using OrthoFinder v2.5.2 with default settings (Emms and Kelly 2019). All SCOs were individually aligned using MAFFT v.7.407 (Katoh and Standley 2013) before trimming with trimAL v.1.4.1 using a gap threshold of 0.1 (Capella-Gutiérrez et al. 2009). The individual SCO alignments were concatenated into a supermatrix using the script geneSticher.py (Schluter 2016), which also produces a partition file. Phylogenetic inference was performed with IQ-TREE v.2.0 running 1000 bootstrap replicates. To evaluate evolutionary relationships on a coalesce theory framework, we used ASTRAL-III v.5.7.3 (Zhang et al. 2018). For this analysis, single gene trees were generated using IQ-TREE v.2.0 and the best-fitting model option (-MFP) with 100 bootstrap iterations (Minh et al. 2020).

### SNP calling analyses

Paired-end reads for all nuclei from each of the four *Funneliformis* spore samples (two cultivated and two collected from the field) were mapped against their respective reference genome assemblies using the BWA-MEM tool of the Burrows-Wheeler aligner (BWA) v.0.7.17 (Li and Durbin 2009) package. SAMtools v.1.14 (Li et al. 2009) was used to convert SAM files to BAM files which were subsequently marked for duplicates, sorted by coordinates, and their read groups replaced using Picard v.2.23.4 (http://broadinstitute.github.io/picard/). SNPs were then called in individual nuclei using the Genome Analysis Toolkit (GATK) v.4.2.5.0 (McKenna et al. 2010) package. The HaplotypeCaller tool was first used to generate general variant calling files (gvcf) for each nucleus with the following parameters: ploidy = 1; minimum-mapping-quality = 30; minimum base quality = 20; max-alternate-alleles = 1. The gvcf files were merged into one gvcf file using the CombineGVCFs tool and subsequently genotyped with the GenotypeGVCFs tool. SNPs were hard-filtered using using GATK’s recommended thresholds: QD, < 2; FS, > 60; MQ, < 40; MQRankSum, < − 12.5; ReadPosRankSum, < − 8; QUAL, < 30; SOR, > 3.0. An additional filtering was applied where sites in nuclei with less than five reads and allele fractions of more than 0.1 but less than 0.9 were marked as missing data, and sites across nuclei with more than 33% missing data were removed using a customized script (SNP_filter.R) available in https://github.com/drowl001/SNP_analysis. SNP densities were calculated for each strain as the number of SNPs in the non-repeat portions of the genome divided by the total size of the non-repeat portion of the genome assembly.

## Results

A *F. geosporum* morpho-species was identified among AM fungal spores extracted from soil associated with a *Potentilla* sp. specimen (Fig. S1), and two representative spores were selected for genome sequencing. Both genome assemblies were over 130 Mb, with approximately 20,000 predicted genes and an estimated BUSCO completeness of 93% (Table 1). The MAT-locus could not be confidently identified in the *Funneliformis* genome assemblies. We determined one of the flanking gene regions, a choline transporter-like homologue, in all the reference assemblies, but single nuclei genome assemblies were fragmented and only a few contigs including the putative choline transporter-like region were recovered. Thus, with the available data we could not determine if the strains are homokaryotic or heterokaryotic for the MAT-locus.

**Table 1:** Genome assembly statistics for two spores of *Funneliformis geosporum* (field collected spores A and B) collected from soil attached to a specimen of *Potentilla* sp. from the Kungsängen Nature Reserve, Uppsala, Sweden

**Species (ID)**	**Size (Mb)**	**Number of contigs**	**N50**	**Largest contig (kb)**	**GC (%)**	**BUSCO (%)**	**Number of genes**	**Repeats (Mb)**
*F. geosporum* (A)	134.3	27,829	10,561	93,301	26.71	C: 93.1 (S:92.8, D:0.3), F:1.4	20,100	68.22
*F. geosporum* (B)	132.9	28,058	10,159	168,938	26.74	C: 93.1 (S:93.1, D:0.0), F:1	20,745	65.51

*C* completeness includes both single copy (S) and duplicated (D) genes, *F* fragmented. Given in % out of 290 BUSCO genes

Extracted SSU regions from the two genome assemblies were identical, and phylogenetic analysis, including published SSU sequences, firmly placed the spores in the genus *Funneliformis*, but identification to species level could not be achieved from the SSU dataset (Fig. S2). The phylogenetic analyses using the rDNA LSU D1 region included six species in the genus *Funneliformis* and despite slightly different LSU sequences, both spores were strongly supported as *F. geosporum* (Fig. S3) as expected from the morphological observations. For TOR2 and FOX2, identical sequences were extracted from the two genome assemblies. TOR2 analysis provided enough resolution to separate *F. geosporum* from sister species in the genus (Fig. S4). Whereas the phylogenetic signal in FOX2 was less clear at the genus level, and did not resolve *F. geosporum* and *F. caledonius* as monophyletic lineages. It did however group the sequences from the field collected spores in *F. geosporum* (Fig. S5). Based on the combined results, we conclude that the sequenced spores from the field represent *F. geosporum*.

**Fig. 1:**
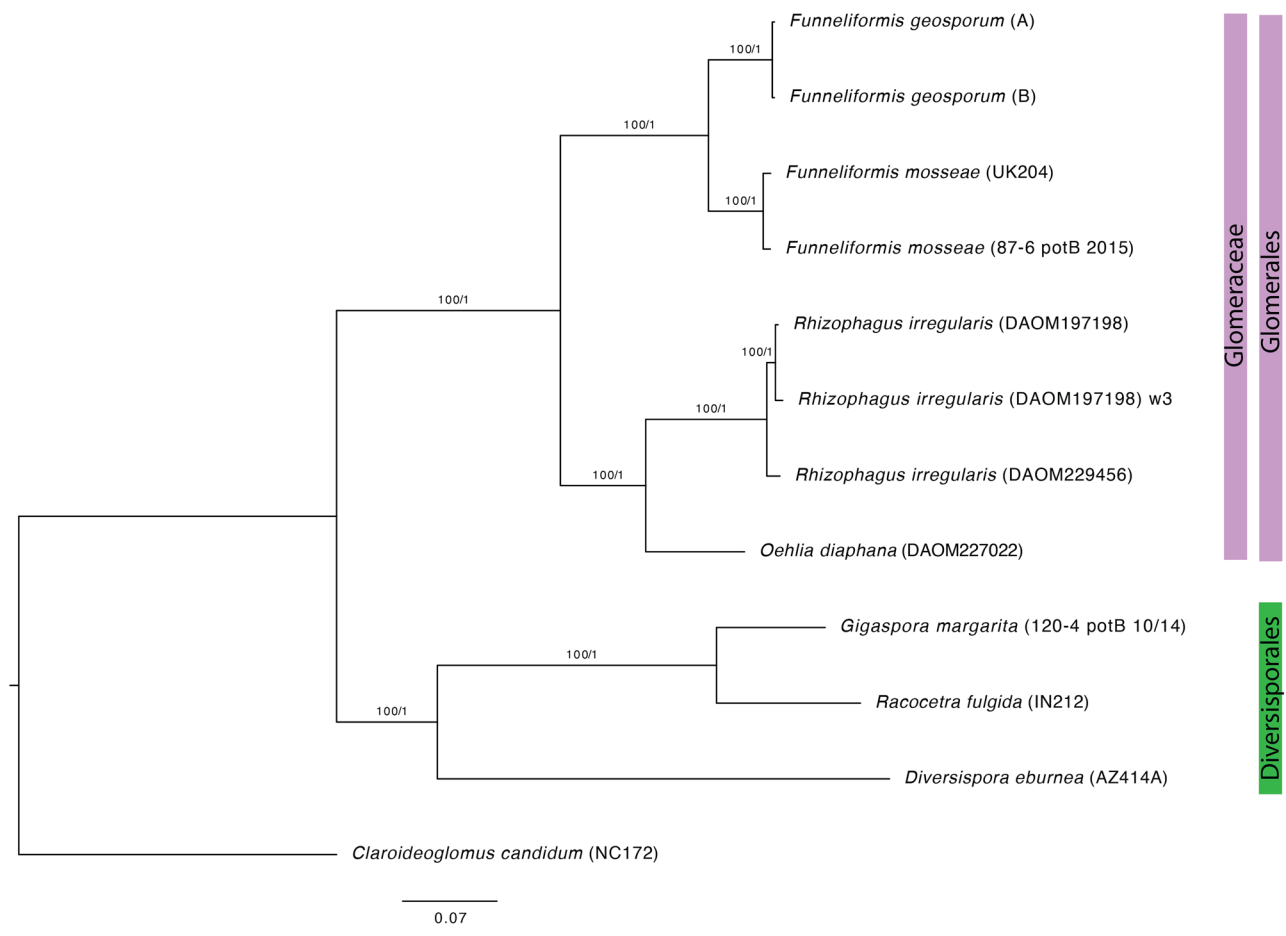
Phylogeny of eight taxa from three genera in Glomerales, three taxa representing the order Diversisporales and *Claroideoglomus candidum* as outgroup. Best maximum likelihood IQ-TREE tree from a concatenated alignment of 1205 single copy orthologs (SCOs) shared among all taxa. The same topology was recovered in an ASTRAL analysis based on individual gene trees for the same SCOs. Multi-locus bootstrapping and local posterior probabilities are indicated at the nodes (MLBS/LPP). All nodes have 100 bootstrap supports based on 100 replications and posterior probability of 1. *Funneliformis geosporum* A and B refer to field collected spores

Here we present genome assemblies from the two spores of *F. geosporum* extracted directly from field soil. This is the first direct genome sequencing of AM fungal spores sampled from a natural habitat. In a phylogenomic analysis that included members of Glomerales and a total of 1205 SCOs present in all taxa, both genomes of *F. geosporum* (field collected spores A and B) were recovered in a well-supported clade together with two strains of *F. mosseae* (Fig. 1). Our analyses demonstrate that *Funneliformis* is the sister genus to *Rhizophagus* and *Oehlia*. The latter is represented by only one species, *O. diaphana*, but the status as a distinct genus is well supported by its separation from *Rhizophagus* on a long branch (Fig. 1), and by morphological synapomorphies (Błaszkowski et al. 2018).

**Fig. 2:**
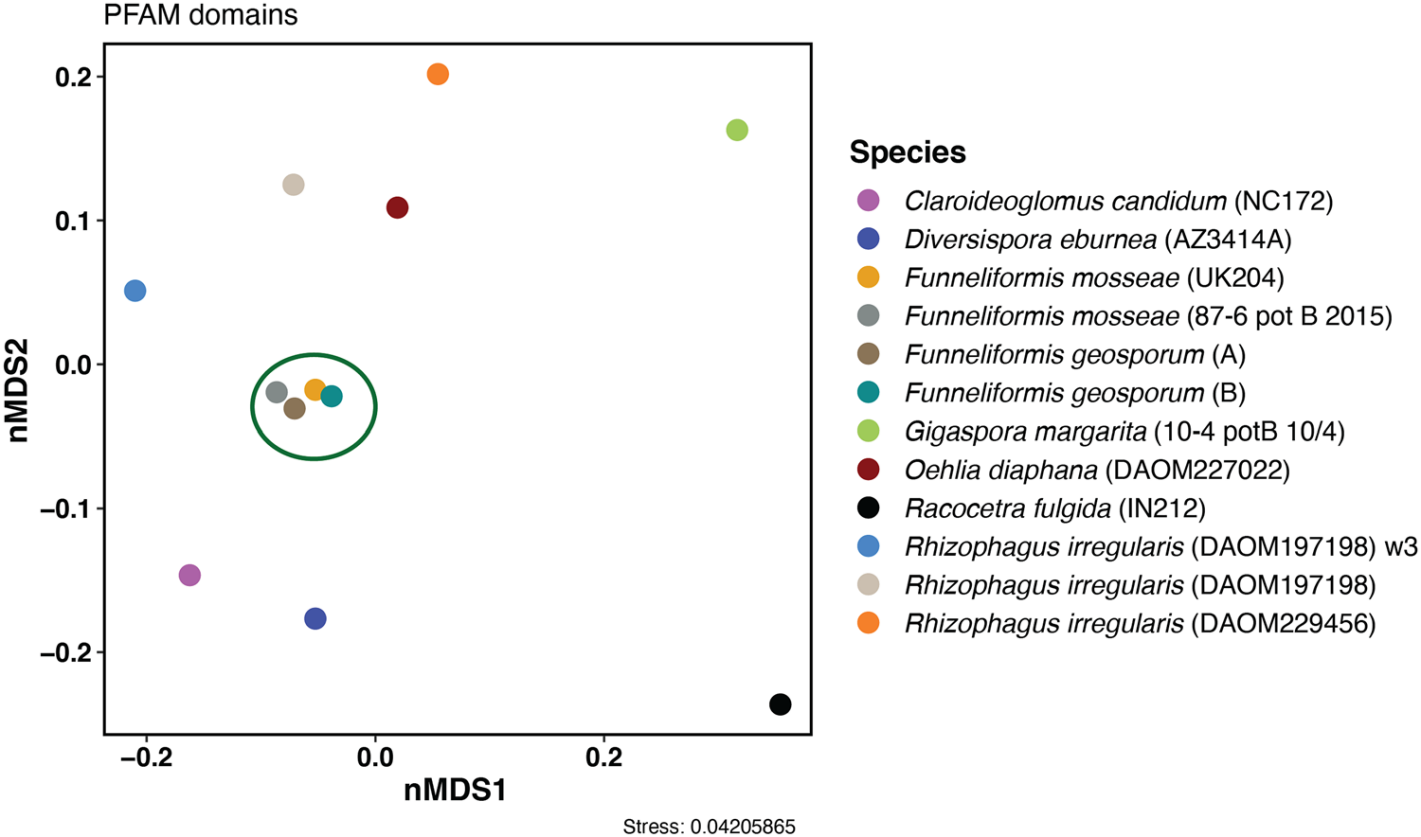
Non-metric multi-dimensional scaling (nMDS) based on functional domain annotations on relative abundance data of all Pfam domains using Bray–Curtis dissimilarity. *Funneliformis* taxa are highlighted with a green circle. *Funneliformis geosporum* A and B refer to field collected spores

**Fig. 3:**
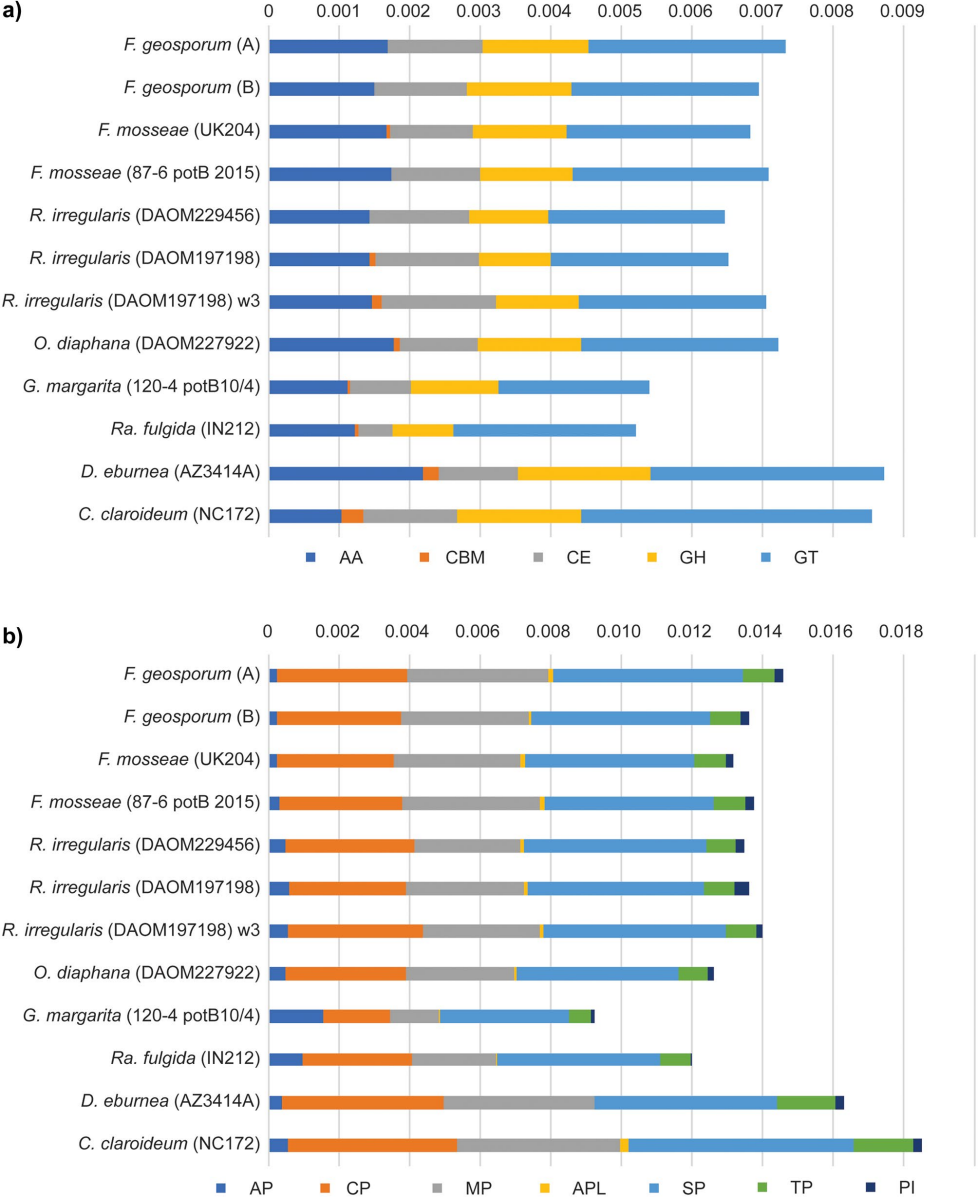
Genes across five carbohydrate-active enzyme (CAZyme) gene families (**a**) and seven peptidases gene families (MEROPS) (**b**), expressed as proportions of the total number of predicted genes from each genome assembly. CAZyme gene families in **a** include Auxiliary activities (AA), Carbohydrate binding modules (CBM), Carbohydrate esterase (CE), Glycoside hydrolase (GH), and Glycosyl transferases (GT). Note that no genes in the Polysaccharide lysases family was detected across the analyzed genome assemblies. The MEROPS gene families in **b** include Aspartic Peptidase (AP), Cysteine peptidase (CP), Metallo peptidase (MP), Asparagine peptide lyase (APL), Serine peptidase (SP), Threonine peptidase (TP), and Protease inhibitors (PT). *Funneliformis geosporum* A and B refer to field collected spores

Comparative genome analyses across the AM fungi included here demonstrate highly similar gene content among strains in the genus *Funneliformis*. Ordination analyses of the Pfam domains show that all *Funneliformis* strains cluster together separated from other taxa (Fig. 2). Curiously, the three genome assemblies of *R. irregularis*, including two of the strain DAOM197198, do not form a similarly tight cluster despite representing a single species (Fig. 2). An analysis of the number of genes across CAZyme gene families revealed high similarity in content across genomes in the genus *Funneliformis* (Fig. 3a, Table S4). None of the analyzed AM fungal genomes contained genes that belong to the Polysaccharide Lysase (PL) family. Few genes in the Carbohydrate Binding Module family (CBM) were found, with the highest record of five genes detected in the outgroup *C. candidum*. Across *Funneliformis*, only one of the *F. mosseae* strains (UK204) contained a gene annotated as member of the CBM family (Fig. 3a). Relative numbers of MEROPS gene families are highly comparable across *Funneliformis* and *Rhizophagus*. However, *Rhizophagus* had more genes in the Aspartic Peptidase (A) family (11–15 genes per genome assembly) compared to *Funneliformis* (5–6 genes per genome assembly) (Fig. 3b). The percentage of genes classified as secreted proteins varied across the analyzed AM fungi, from 0.4% in *G. margarita* to 3.8% in *Ra. fulgida* (Fig. S6). Corresponding numbers across strains in the genus *Funneliformis* were highly comparable with an average of 2.8%. Slightly higher values were obtained for *Rhizophagu*s with an average of 3.6% of all genes being classified as secreted proteins (Fig. S6). None of the comparative genome analyses suggests any major differences in gene content composition for members of the genus *Funneliformis* whether isolated from field collected or cultured material.

All *Funneliformis* strains generally showed a low intra-isolate genetic variation, with SNP densities ranging from 0.04 to 0.26 SNPs per kilobase in the non-repeat regions of the genome. The two *F. geosporum* strains show a higher within-strain variation with SNP densities at 0.24 and 0.26 SNPs/kb, compared to the two *F. mosseae* strains at 0.04 and 0.13 SNPs/kb respectively (Table 2).

**Table 2:** SNP statistics for two published strains of *F. mosseae* and two field collected spores (A and B) of *F. geosporum*

	***F. mosseae*** **UK204**	***F. mosseae*** **87–6 pot B 2015**	***F. geosporum* (A)**	***F. geosporum* (B)**
Total number of SNPs	84,819	46,678	110,604	94,216
Number of SNPs non-rep	9808	2899	17,404	15,370
Number of SNPs coding region	2563	967	7184	5934
Density SNP/kb^*^	0.13	0.04	0.26	0.24

^*^Density calculated as the number of SNPs in the non-repeat region of the genome divided by the size of the non-repeat region of the genome assembly

## Discussion

Our work demonstrates that genome assemblies can be generated from AM fungus spores collected in the field. The study was possible thanks to development of a workflow from single nuclei sorting through whole genome amplification and sequencing to a curated assembly pipeline (Montoliu-Nerin et al. 2020). Phylogenetic analysis of the two spores placed them firmly in the genus *Funneliformis* as *F. geosporum* which is in accordance with the morphological characterization at the time of sampling. The number of predicted genes observed in the two genome assemblies from *F. geosporum* spores is very similar and of a quality well suited for phylogenomic analysis. Extraction of marker genes such as D1 of LSU, TOR2, and FOX2, followed by focused phylogenetic analysis with known references, proved to be an efficient method for species identification. While slightly different LSU sequences were extracted, the TOR2 and FOX2 sequence were identical for the two spores (Fig. S3–S5). This is probably because the rDNA operon occurs as multiple separate copies in AM fungi (Maeda et al. 2018) and different variants were likely assembled in this study. FOX2 and TOR2 on the other hand are single copy genes.

In addition to the field collected *F. geosporum* spores sequenced in this study, our analysis included two *F. mosseae* strains (Montoliu-Nerin et al. 2021). Of these, the strain *F. mosseae* 87–6 potB 10/4 has been in culture since 2003 while the strain *F. mosseae* UK204 has been in culture since 1994. Assembly statistics (Table 1, Table S3) and genome annotation comparisons (Fig. 2, S6) show no indication of differences in genome content between taxa harvested directly from the field compared to taxa maintained in culture for many generations. Nevertheless, it is possible that other aspects of genome organization not covered by gene annotations could differ between cultured and field collected strains. For instance, considerable chromosome length variation and structural rearrangements have been observed across three well studied strains of *R. irregularis* separately isolated from the same field (Yildirir et al. 2022). Furthermore, we acknowledge that an appropriate comparison of genetic variation would require that field collected and cultured strains of the same species are analyzed.

While all four *Funneliformis* strains cluster tightly in the ordination analysis of functional domains, the three assemblies of *R. irregularis* were far more dispersed in the two visualized dimensions (Fig. 2). This might be because of large variation in gene content among *Rhizophagus* strains, where large copy number variation has been reported (Mathieu et al. 2018). It also might be due to differences in the quality of the genome assemblies and annotations (Table S3). Genome assemblies generated from single amplified and sequenced nuclei are notably fragmented, with a N50 of 21,000 kb compared to 51,000 kb (Chen et al. 2018) and 137,000 (Morin et al. 2019) for assemblies generated from high-quality DNA extracts. With decreasing fragmentation, more genes can be properly predicted and annotated potentially affecting the results of overall protein family analyses (Fig. 3). However, the *R. irregularis* assembly generated from amplified and sequenced nuclei has been shown to be highly similar to a conventionally sequenced genome of the same strain (Montoliu-Nerin et al. 2021), and it is likely that there is a strong biological signal in the observed differences in functional domain composition within the two genera in Glomeraceae.

Analysis of SNP density suggests a higher genetic diversity for the field collected spores of *F. geosporum* (0.24–0.26 SNPs/kb) than for the cultured strains (0.04–0.13 SNPs/kb) of *F. mosseae*. The numbers are comparable to published SNP density estimates from other cultured AM fungus strains. SNP densities around 0.2 SNPs/kb have been estimated in *R. irregularis* strains that are homokaryons for the MAT-locus, while strains that are heterokaryons for the MAT-locus have higher densities from 0.45 to 0.8 SNPs/kb (Ropars et al. 2016). Observed differences in SNP density between genome assemblies of *F. geosporum* and *F. mosseae* may reflect differences in genome organization with regard to the mating type of nuclei in the strains. In the current study, however, we could not determine if the analyzed genome assemblies represent strains that are homokaryotic or heterokaryotic for the MAT-locus. This will be possible to resolve in the future when the MAT-locus is identified in *Funneliformis*.

In conclusion, a single-nucleus sequencing approach to assemble the whole genome of spores directly obtained from the field opens the door for extensive genome sampling of hitherto un-sequenced diversity among AM fungi. For instance, genomes could be assembled for species that are abundantly detected in undisturbed natural settings but cannot be grown in culture (Ohsowski et al. 2014). Furthermore, the method could facilitate population genetic studies of natural populations by extracting marker genes from genome assemblies representing different spores or could enable estimating genetic variation within spores to refine our understanding of how geographic distance and agricultural practices affect the population structure of AM fungi (Rosendahl and Matzer 2008; Rosendahl et al. 2009).

## Supplementary Information

Below is the link to the electronic supplementary material.

Supplementary file1 (XLSX 935 KB)

Supplementary file2 (DOCX 2777 KB)

## Data Availability

Raw reads, assemblies, and annotations are available at ENA with the accession number PRJEB55531. Supporting data including alignments and phylogenetic trees are available in Figshare 10.17044/scilifelab.20120150.
