# Supplementary material for: Whole genome analyses based on single, field collected spores of the arbuscular mycorrhizal fungus Funneliformis geosporum
Source: Mycorrhiza. 2022 Sep 26;32(5-6):361–71. doi: 10.1007/s00572-022-01091-4 (PMC9560946; doi:10.1007/s00572-022-01091-4)
Supplement: Supplementary file 2 — Supplementary file2 (DOCX 2777 KB) [file 572_2022_1091_MOESM2_ESM.docx]

**Whole genome analyses based on single, field collected spores of the arbuscular mycorrhizal fungus *Funneliformis geosporum***

Shadi Eshghi Sahraei^1*^, Marisol Sánchez-García^1,2^, Mercè Montoliu-Nerin^1,3^, David Manyara^1^, Claudia Bergin^4^, Søren Rosendahl^5^, Anna Rosling^1*^

^1^ Department of Ecology and Genetics, Uppsala University, Uppsala, Sweden

^2^ Uppsala Biocentre, Department of Forest Mycology and Plant Pathology, Swedish

University of Agricultural Sciences, Uppsala, Sweden

^3^ Department of Cell and Molecular Biology, Uppsala University, Uppsala, Sweden

^4^ Microbial Single Cell Genomics Facility, Department of Cell and Molecular Biology, Science for Life Laboratory, Uppsala University, Uppsala, Sweden

^5^ Department of Biology, University of Copenhagen, Copenhagen, Denmark

* Corresponding authors: [Anna.Rosling@ebc.uu.se](mailto:Anna.Rosling@ebc.uu.se) ; shadi.eshghi.sahraei@ebc.uu.se

**Supplementary table 1**. Genome statistics for all single nucleus assemblies in each field collected spore, *Funneliformis geosporum* (A, B) and previously published *Funneliformis mosseae* (UK204, 87-6 pot B 2015).

| ***F. geosporum* (A)** **Nucleus (id)** | **Size (Mb)** | **Number of contigs** | **N50** | **Largest contig (kb)** | **GC (%)** | **BUSCO (%)^*^** |
| --- | --- | --- | --- | --- | --- | --- |
| 1 | 68.25 | 20,724 | 6,793 | 75.24 | 27.16 | C: 45.2 (S: 44.9, D: 0.3), F: 7.5 |
| 2 | 56.76 | 17,688 | 6,680 | 61.19 | 27.3 | C: 39.3 (S: 39.2, D: 0.1), F: 8.3 |
| 3 | 22.24 | 7,720 | 5,256 | 36.17 | 27.99 | C: 12.4 (S: 12.4, D: 0.0), F: 4.7 |
| 4 | 20.59 | 7,605 | 4,767 | 39.12 | 27.72 | C: 11.3 (S: 11.2, D: 0.1), F: 4.0 |
| 5 | 37.74 | 11,285 | 6,621 | 54.86 | 27.29 | C: 26.2 (S: 26.1, D: 0.1), F: 4.0 |
| 6 | 30.28 | 10,791 | 5,548 | 50.98 | 27.62 | C: 18.6 (S: 18.6, D: 0.0), F: 5.0 |
| 7 | 51.55 | 16,254 | 6,751 | 58.66 | 27.22 | C: 35.2 (S: 35.1, D: 0.1), F: 5.9 |
| 8 | 13.92 | 4,987 | 4,664 | 35.6 | 28.27 | C: 8.4 (S: 8.3, D: 0.1), F: 3.3 |
| 9 | 8.50 | 4,099 | 2,940 | 27.18 | 28.31 | C: 2.6 (S: 2.6, D: 0.0), F: 2.0 |
| 11 | 21.2 | 7,165 | 5,243 | 73.84 | 27.83 | C: 14.5 (S: 14.4, D: 0.1), F: 5.3 |
| 13 | 56.20 | 23,490 | 4,292 | 39.65 | 27.3 | C: 34.0 (S: 33.5, D: 0.5), F: 9.4 |
| 14 | 37.76 | 12,133 | 6,527 | 57.38 | 27.44 | C: 28.2 (S: 28.2, D: 0.0), F: 6.2 |
| 15 | 34.54 | 11,476 | 5,680 | 47.18 | 27.67 | C: 23.5 (S: 23.4, D: 0.1), F: 5.8 |
| 16 | 52.31 | 21,555 | 4,678 | 47.19 | 27.46 | C: 32.7 (S: 32.7, D: 0.0), F: 7.0 |
| 17 | 10.32 | 4,546 | 3,384 | 20.42 | 28.25 | C: 14.5 (S: 14.4, D: 0.1), F: 5.3 |
| 18 | 36.1 | 12,117 | 6,288 | 62.72 | 27.43 | C: 22.7 (S: 22.6, D: 0.1), F: 5.0 |
| 19 | 52.33 | 16,113 | 6,751 | 55.71 | 27.34 | C: 35.9 (S:35.6, D:0.3), F:6.6 |
| 20 | 45.67 | 14,748 | 6,580 | 74.82 | 27.33 | C: 34.9 (S: 34.8, D: 0.1), F: 5.3 |
| 21 | 34.12 | 10,577 | 6,173 | 48.73 | 27.49 | C: 24.7 (S: 24.7 D: 0.0), F: 4.5 |
| 22 | 19.7 | 5,868 | 6,797 | 50.9 | 27.56 | C: 12.1 (S: 12.1, D: 0.0), F: 2.2 |
| 23 | 14.45 | 6,684 | 3,129 | 24.82 | 28.32 | C: 6.7 (S: 6.7, D: 0.0), F: 3.8 |
| 24 | 19.70 | 6,691 | 6,468 | 61.0 | 27.7 | C: 13.7 (S: 13.7, D: 0.0), F: 3.3 |

| ***F. geosporum (B)*** **Nucleus (id)** | **Size (Mb)** | **Number of contigs** | **N50** | **Largest contig (kb)** | **GC (%)** | **BUSCO (%)^*^** |
| --- | --- | --- | --- | --- | --- | --- |
| 1 | 86.9 | 26,995 | 6,562 | 80.98 | 27.14 | C: 62.0 (S: 61.6, D: 0.4), F: 7.3 |
| 2 | 20.85 | 7,013 | 5,086 | 35.45 | 27.84 | C: 13.8 (S: 13.7, D: 0.1), F: 5.0 |
| 3 | 50.48 | 17,688 | 5,705 | 85.91 | 27.58 | C: 34.3 (S: 34.0, D: 0.3), F: 6.3 |
| 4 | 35.4 | 16,816 | 3,289 | 31.86 | 27.56 | C: 16.0 (S: 16.0, D: 0.0), F: 6.6 |
| 5 | 8.92 | 4,311 | 2,916 | 20.53 | 29.19 | C: 4.1 (S: 4.0, D: 0.1), F: 1.7 |
| 6 | 6.97 | 3,526 | 2,688 | 16.43 | 28.14 | C: 3.0 (S: 3.0, D: 0.0), F: 2.2 |
| 7 | 64.61 | 19,372 | 6,662 | 51.84 | 27.21 | C: 36.7 (S: 36.4, D: 0.3), F: 6.3 |
| 8 | 19.12 | 6,792 | 4,622 | 37.12 | 28.01 | C: 11.9 (S: 11.9, D: 0.0), F: 3.8 |
| 9 | 30.58 | 9,590 | 5,869 | 35.2 | 27.48 | C: 22.4 (S: 22.4, D: 0.0), F: 5.3 |
| 10 | 38.38 | 12,634 | 5,749 | 52.03 | 27.43 | C: 23.4 (S: 23.4, D: 0.0), F: 4.7 |
| 11 | 12.51 | 4,730 | 4,256 | 31.71 | 28.38 | C: 7.1 (S: 7.0, D: 0.1), F: 2.8 |
| 12 | 46.84 | 16,276 | 5,352 | 57.53 | 27.50 | C: 27.8 (S: 27.8, D: 0.0), F: 6.1 |
| 13 | 3.23 | 1,260 | 3,907 | 26.3 | 28.29 | C: 0.9 (S: 0.9, D: 0.0), F: 1.1 |
| 14 | 55.93 | 17,599 | 6,793 | 58.08 | 27.16 | C: 39.4 (S: 39.1, D: 0.3), F: 7.4 |
| 15 | 25.94 | 9,629 | 4,561 | 35.14 | 27.90 | C: 15.7 (S: 15.7, D: 0.0), F: 4.2 |
| 16 | 37.9 | 12,964 | 5,529 | 44.63 | 27.48 | C: 24.8 (S: 24.7, D: 0.1), F: 5.8 |
| 17 | 33.23 | 10,866 | 5,903 | 54.94 | 27.53 | C: 21.4 (S: 21.1, D: 0.3), F: 4.2 |
| 18 | 17.8 | 6,712 | 4,317 | 36.4 | 28.01 | C: 9.8 (S: 9.8, D: 0.0), F: 3.4 |
| 19 | 18.47 | 6,852 | 4,517 | 34.61 | 28.01 | C: 10.0 (S: 10.0, D: 0.0), F: 3.7 |

| ***F. mosseae* (UK204)**  **Nucleus** | **Size (Mb)** | **Number of contigs** | **N50** | **Largest contig (kb)** | **GC (%)** | **BUSCO (%)^*^** |
| --- | --- | --- | --- | --- | --- | --- |
| 1 | 27.23 | 18,248 | 1,966 | 18.25 | 27.93 | C: 6.7 (S: 5.9, D: 0.8 ), F: 3.7 |
| 2 | 6.69 | 7,534 | 808 | 10.47 | 29.87 | C: 0.4 (S: 0.4, D: 0.0 ), F: 1.1 |
| 3 | 24.43 | 15,430 | 2,182 | 15.49 | 27.61 | C: 4.6 (S: 4.2, D: 0.4 ), F: 3.3 |
| 4 | 31.58 | 19,542 | 2,222 | 22.7 | 27.40 | C: 7.0 (S: 6.2, D: 0.8 ), F: 3.7 |
| 5 | 40.43 | 26,603 | 2,011 | 12.38 | 28.61 | C: 10.6 (S: 9.5, D: 1.1 ), F: 7.3 |
| 6 | 53.12 | 29,793 | 2,577 | 19.91 | 27.33 | C: 10.0 (S: 9.1, D: 0.9 ), F: 6.7 |
| 7 | 37.68 | 23,565 | 2,149 | 18.06 | 27.45 | C: 5.8 (S: 5.4, D: 0.4 ), F: 6.2 |
| 8 | 50.95 | 31,051 | 2,265 | 19.36 | 27.74 | C: 8.7 (S: 7.9, D: 0.8 ), F: 6.3 |
| 9 | 30.81 | 19,774 | 2,084 | 17.67 | 28.18 | C: 6.7 (S: 5.8, D: 0.9), F: 5.7 |
| 10 | 72.73 | 44,686 | 2,227 | 18.22 | 28.15 | C: 17.7 (S: 15.6, D: 2.1), F: 11.2 |
| 11 | 25.51 | 18,113 | 1,802 | 10.9 | 28.52 | C: 4.6 (S: 4.1, D: 0.5), F: 4.6 |
| 12 | 28.18 | 17,716 | 2,161 | 17.61 | 28.14 | C: 5.1 (S: 4.7, D: 0.4), F: 4.7 |
| 13 | 18.1 | 12,246 | 1,961 | 15.64 | 27.44 | C: 4.4 (S: 3.7, D: 0.7), F: 2.4 |
| 14 | 93.73 | 51,270 | 2,777 | 30.72 | 27.75 | C: 31.6 (S: 28.8, D: 2.8), F: 11.6 |
| 15 | 26.84 | 18,699 | 1,858 | 11.82 | 28.06 | C: 4.8 (S: 4.1, D: 0.7), F: 3.4 |
| 16 | 48.05 | 26,474 | 2,719 | 32.62 | 27.35 | C: 14.0 (S: 13.1, D: 0.9), F: 5.4 |
| 17 | 28.5 | 20,705 | 1,777 | 12.5 | 28.83 | C: 6.4 (S: 5.7, D: 0.7), F: 6.1 |
| 18 | 14.41 | 6,658 | 3,701 | 36.38 | 29.37 | C: 4.4 (S: 4.1, D: 0.3), F: 1.5 |
| 19 | 7.02 | 8,506 | 774 | 8.83 | 29.90 | C: 0.2 (S: 0.1, D: 0.1), F: 0.7 |
| 20 | 41.25 | 25,665 | 2,193 | 19.23 | 27.70 | C: 7.1 (S: 6.3, D: 0.8), F: 5.4 |
| 21 | 41.79 | 26,975 | 2,069 | 13.1 | 28.14 | C: 6.2 (S: 5.3, D: 0.9), F: 6.1 |
| 22 | 10.37 | 10,416 | 980 | 8.4 | 29.89 | C: 1.7 (S: 1.6, D: 0.1), F: 1.7 |
| 23 | 34.3 | 24,437 | 1,773 | 12.14 | 27.32 | C: 4.6 (S: 4.1, D: 0.5), F: 4.9 |
| 24 | 13.08 | 10,803 | 1,484 | 11.21 | 28.44 | C: 1.8 (S: 1.5, D: 0.3), F: 2.2 |

| ***F. mosseae* (87-6 pot B 2015)**  **Nucleus** | **Size (Mb)** | **Number of contigs** | **N50** | **Largest contig (kb)** | **GC (%)** | **BUSCO (%)^*^** |
| --- | --- | --- | --- | --- | --- | --- |
| 1 | 51.97 | 21,930 | 4,367 | 47.37 | 26.43 | C: 19.9 (S: 17.9, D: 2.0), F: 8.8 |
| 2 | 34.07 | 19,465 | 2,637 | 27.93 | 26.47 | C: 10.2 (S: 9.1, D: 1.1), F: 4.7 |
| 3 | 26.30 | 13,608 | 3,154 | 24.72 | 26.39 | C: 8.2 (S: 7.5, D: 0.7), F: 4.4 |
| 4 | 69.38 | 34,137 | 3,406 | 29.18 | 26.27 | C: 21.7 (S: 19.9, D: 1.8), F: 8.4 |
| 5 | 2.68 | 2,447 | 1,344 | 10.97 | 30.84 | C: 0.3 (S: 0.3, D: 0.0), F: 0.1 |
| 6 | 5.29 | 4,307 | 1,575 | 8.01 | 28.04 | C: 0.9 (S: 0.8, D: 0.1), F: 1.1 |
| 7 | 26.31 | 12,900 | 3,449 | 22.7 | 26.76 | C: 7.3 (S: 6.5, D: 0.8), F: 4.5 |
| 8 | 8.68 | 5,270 | 2,518 | 18.36 | 27.96 | C: 2.0 (S: 1.7, D: 0.3), F: 2.4 |
| 9 | 32.25 | 14,981 | 3,698 | 39.86 | 26.60 | C: 10.0 (S: 8.8, D: 1.2), F: 3.7 |
| 10 | 74.98 | 34,134 | 3,906 | 38.73 | 26.02 | C: 25.5 (S: 23.1, D: 2.4), F: 8.6 |
| 11 | 29.23 | 17,071 | 2,598 | 19.47 | 26.24 | C: 7.7 (S: 7.3, D: 0.4), F: 4.2 |
| 12 | 23.94 | 13,520 | 2,828 | 21.02 | 26.84 | C: 7.3 (S: 6.6, D: 0.7), F: 3.0 |
| 13 | 47.14 | 22,000 | 3,677 | 33.14 | 26.18 | C: 14.5 (S: 12.5, D: 2.0), F: 5.8 |
| 14 | 3.08 | 3,468 | 774 | 8.23 | 32.00 | C: 0.3 (S: 0.3, D: 0.0), F: 0.4 |
| 15 | 9.11 | 5,136 | 2,788 | 15.38 | 28.50 | C: 2.5 (S: 2.4, D: 0.1), F: 1.6 |
| 16 | 20.51 | 11,264 | 2,929 | 27.09 | 27.73 | C: 4.9 (S: 4.4, D: 0.5), F: 2.1 |
| 17 | 10.64 | 5,348 | 3,504 | 21.29 | 27.30 | C: 3.9 (S: 3.6, D: 0.3), F: 1.8 |
| 18 | 3.81 | 2,633 | 2,256 | 14.10 | 28.79 | C: 0.8 (S: 0.8, D: 0.0), F: 0.5 |
| 19 | 14.37 | 11,393 | 1,634 | 20.58 | 29.80 | C: 2.4 (S: 2.4, D: 0.0), F: 1.6 |
| 20 | 34.0 | 16,461 | 3,663 | 42.65 | 26.31 | C: 10.2 (S: 9.5, D: 0.7), F: 3.8 |

^*^ C: completeness includes both single copy (S) and duplicated (D) genes, F: fragmented. Given in % out of 758 BUSCO genes

**Supplementary table 2**. List of genome assemblies included in the analyses.

| **Species name** | **Strain number** | **Published in** |
| --- | --- | --- |
| *Funneliformis mosseae* | UK204 | Montoliu-Nerin *et al.* 2021 |
| *Funneliformis mosseae* | 87-6 pot B 2015 | Montoliu-Nerin *et al.* 2021 |
| *Funneliformis geosporum* | Field collected spore A | Current study |
| *Funneliformis* *geosporum* | Field collected spore B | Current study |
| *Rhizophagus irregularis* | DAOM197198 | Chen *et al*. 2018 |
| *Rhizophagus irregularis* | DAOM197198 (w3) | Montoliu-Nerin *et al.* 2021 |
| *Rhizophagus irregularis* | DAOM229456* | Morin *et al.* 2019 |
| *Oehlia diaphana* | DAOM227022 | Morin *et al.* 2019 |
| *Gigaspora margarita* | 120-4 potB 10/4 | Montoliu-Nerin *et al.* 2021 |
| *Racocetra fulgida* | IN212 | Montoliu-Nerin *et al.* 2021 |
| *Diversispora eburnea* | AZ3414A | Montoliu-Nerin *et al.* 2021 |
| *Claroideoglomus candidum* | NC172 | Montoliu-Nerin *et al.* 2021 |

* DAOM229456 corresponds to MUCL43196 in Morin et al. 2019

**Supplementary table 3.** Genome statistics for all taxa included in the analysis.

| **Species name** | **Strain number** | **Assembly size (Mb)** | **Number of contigs** | **N50** | **%GC** | **Number of genes** |
| --- | --- | --- | --- | --- | --- | --- |
| *Funneliformis mosseae* | UK204 | 146 | 31,052 | 9,933 | 26 | 20,376 |
| *Funneliformis mosseae* | 87-6 pot B 2015 | 146 | 27,134 | 11,988 | 25 | 19,051 |
| *Funneliformis* *geosporum* | Field collected spore A | 134 | 27,829 | 10,561 | 27 | 20,094 |
| *Funneliformis geosporum* | Field collected spore B | 133 | 28,058 | 10,159 | 27 | 20,732 |
| *Rhizophagus irregularis* | DAOM197198 | 126 | 2,764 | 137,499 | 24 | 23,252 |
| *Rhizophagus irregularis* | DAOM197198 (w3) | 125 | 11,196 | 51,491 | 26 | 26,580 |
| *Rhizophagus irregularis* | DAOM229456 | 116 | 15,939 | 21,163 | 27 | 23,258 |
| *Oehlia diaphana* | DAOM227022 | 137 | 2,592 | 147,873 | 22 | 21,549 |
| *Gigaspora margarita* | 120-4 potB 10/4 | 575 | 173,647 | 5,875 | 27 | 57,019 |
| *Racocetra fulgida* | IN212 | 296 | 105,307 | 4,839 | 28 | 24,442 |
| *Diversispora eburnea* | AZ3414A | 65 | 9,803 | 22,612 | 26 | 13,318 |
| *Claroideoglomus candidum* | NC172 | 68 | 12,232 | 15,216 | 28 | 16,486 |

**Supplementary table 4.** Number of genes across six carbohydrate-active enzyme (CAZyme) gene families in four *Funneliformis* genome assemblies

| **CAZyme families** | ***F. mosseae***  **UK204** | ***F. mosseae***  **87-6 pot B 2015** | ***F. geosporum* (A^*^)** | ***F. geosporum* (B^*^)** |
| --- | --- | --- | --- | --- |
| Auxiliary activity (AA) | 34 | 33 | 34 | 31 |
| Carbohydrate-binding module (CBM) | 1 | 0 | 0 | 0 |
| Carbohydrate esterase (CE) | 24 | 24 | 27 | 27 |
| Glycoside hydrolase (GH) | 27 | 25 | 30 | 31 |
| Glycoside transferase (GT) | 53 | 53 | 56 | 55 |
| Polysaccharide lyase (PL) | 0 | 0 | 0 | 0 |

^*^field collected spore


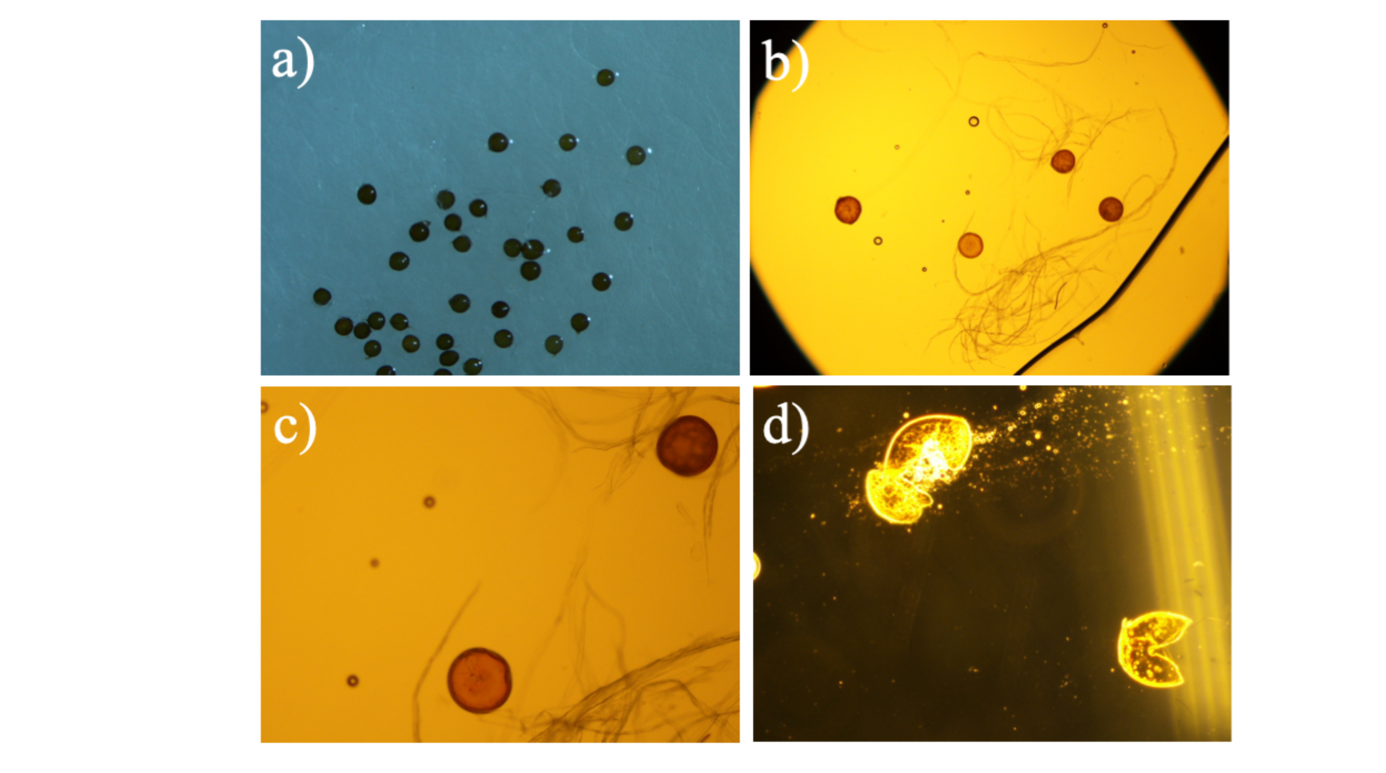


**Figure S1**. Arbuscular mycorrhizal fungal spores identified as *Funneliformis geosporum* after inspection under the stereomicroscope a) placed on moist filter paper. Close up under microscope b) 10x and c) 20x magnification and d) crushed spores under 20x magnification.

**Figure S2**. Best maximum likelihood tree from an alignment of the extracted small subunit (SSU) of the rDNA from the newly assembled genomes of two spores of *Funneliformis geosporum* identified from field samples (field collected spore A and field collected spore B in green) with Glomeromycota SSU sequences published in Krüger *et al*. 2012. Extracted SSU regions from previously published genome assemblies generated with the same method (Montoliu-Nerin *et al.* 2021) were also included in the tree (purple and orange labels). Of these, the ones included in downstream analyses are shown in orange. Branch labels show bootstrap support calculated from 1000 bootstrap replicates. *F.geosporum* A and B refer to field collected spores.


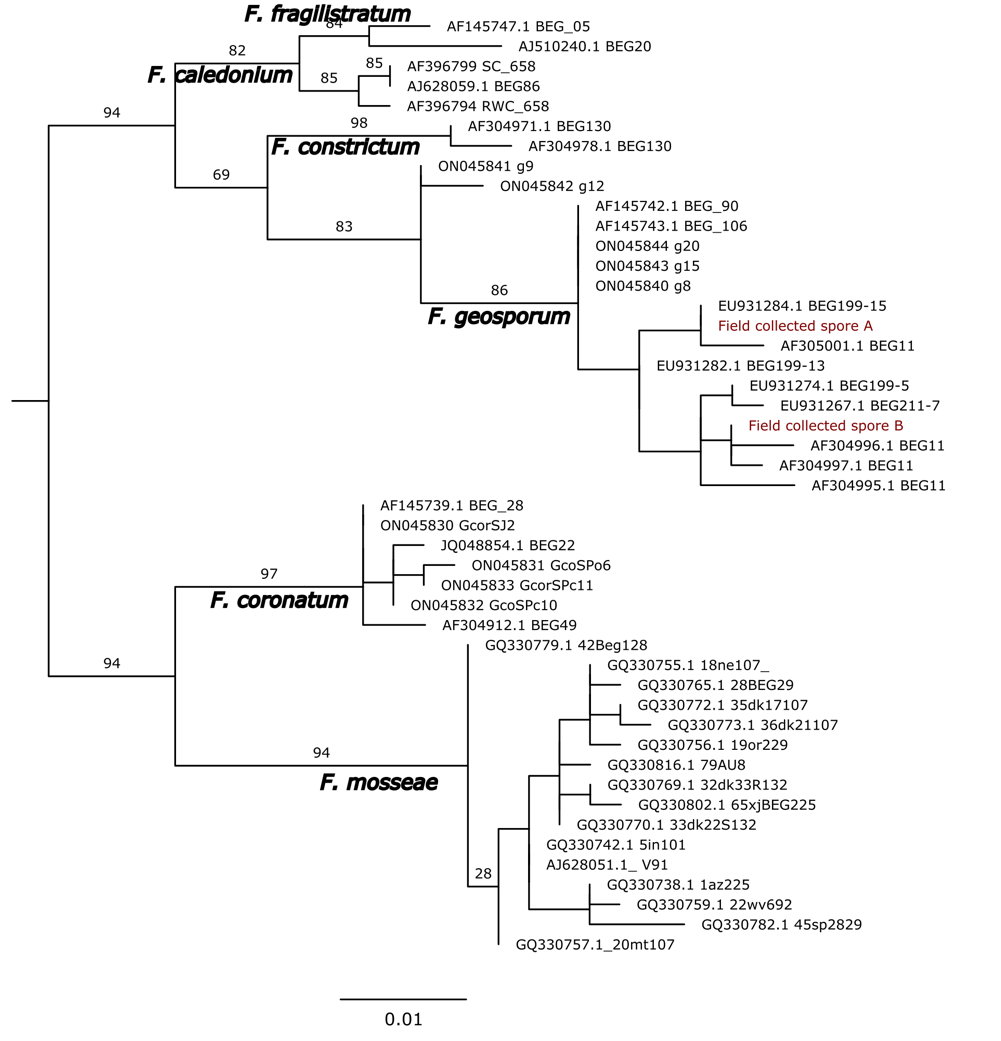


**Figure S3.** Best maximum likelihood tree from and alignment of the D1 region of LSU. Branch labels show bootstrap support calculated from 1000 bootstrap replicates.


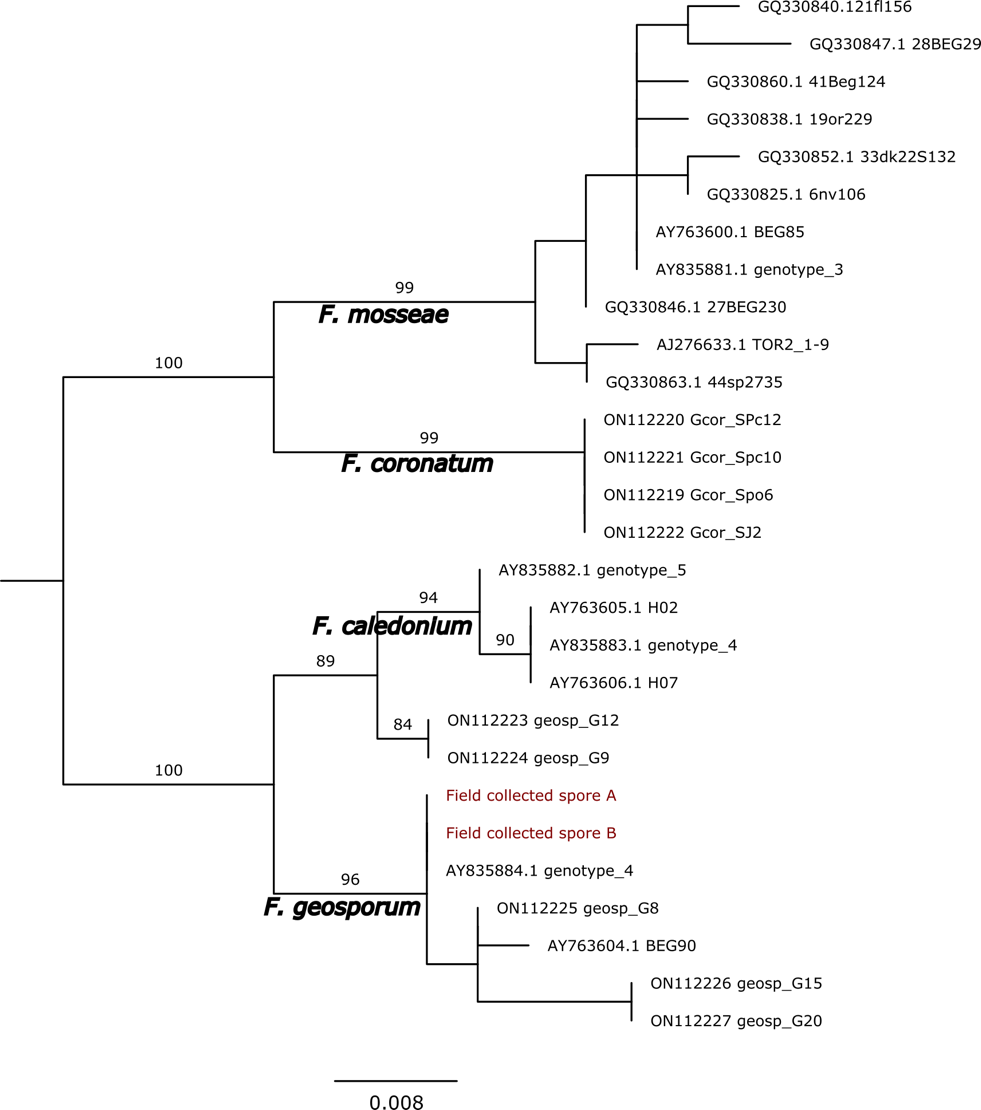


**Figure S4**. Best maximum likelihood tree from and alignment of the TOR2 region. Branch labels show bootstrap support calculated from 1000 bootstrap replicates.


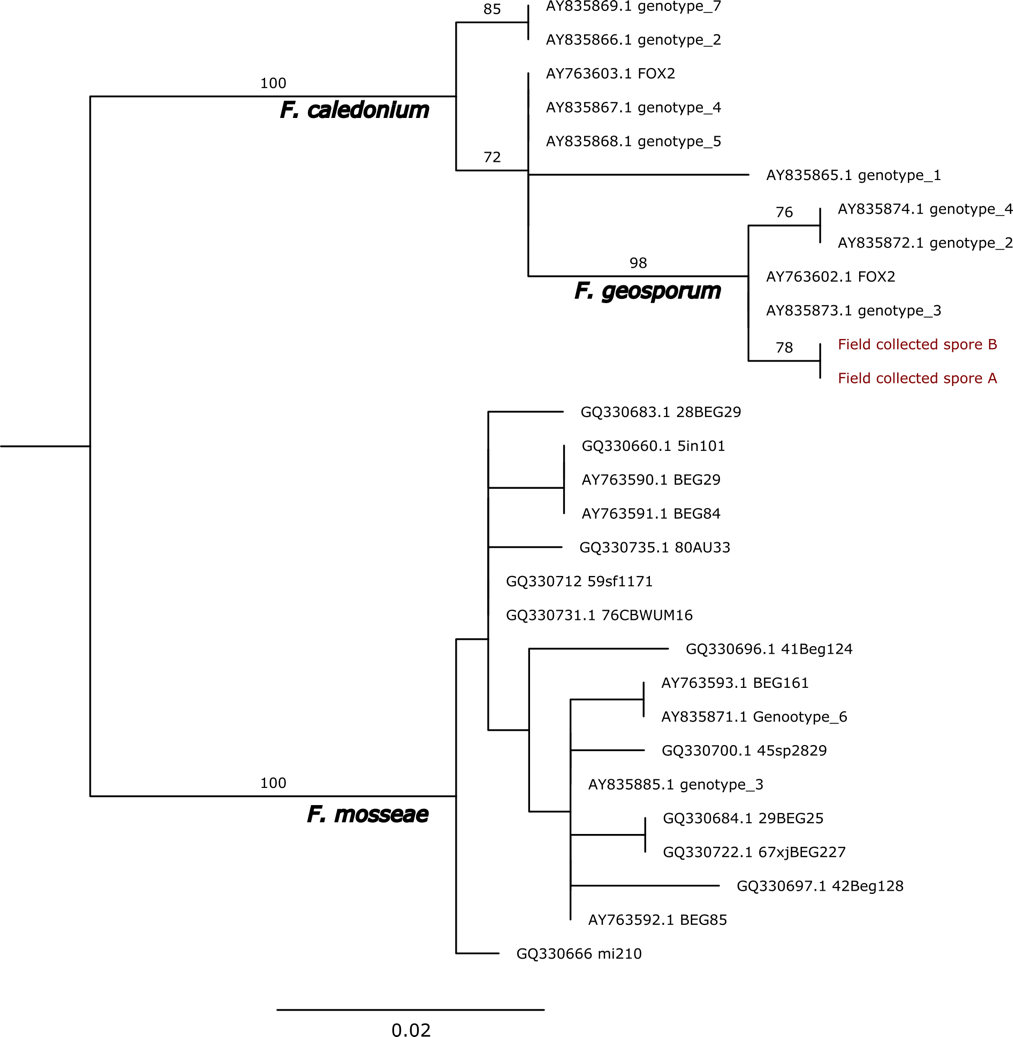


**Figure S5.** Best maximum likelihood tree from and alignment of the FOX2. Branch labels show bootstrap support calculated from 1000 bootstrap replicates.

**
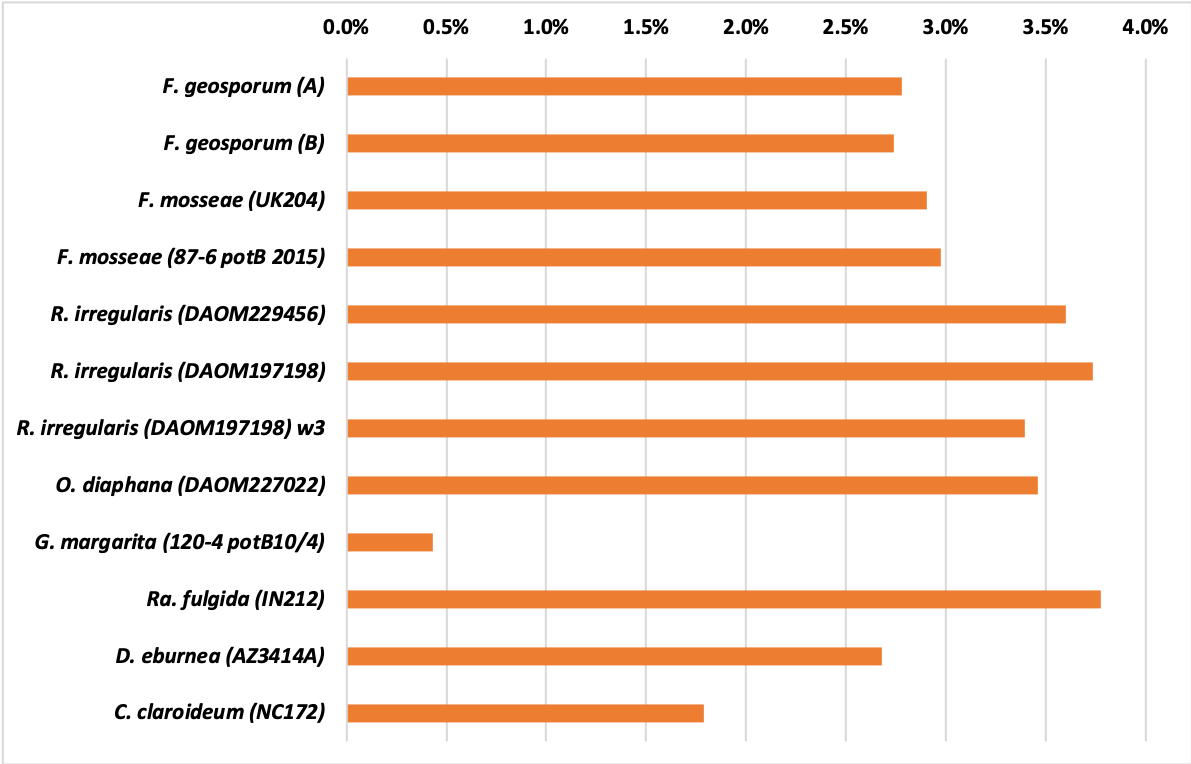
**

**Figure S6.** Percentage of secreted proteins out of all predicted genes across all analyzed arbuscular mycorrhizal taxa. *F.geosporum* A and B refer to field collected spores.
